# The Treatment of Peritoneal Carcinomatosis in Advanced Gastric Cancer: State of the Art

**DOI:** 10.1155/2014/912418

**Published:** 2014-02-17

**Authors:** Giulia Montori, Federico Coccolini, Marco Ceresoli, Fausto Catena, Nicola Colaianni, Eugenio Poletti, Luca Ansaloni

**Affiliations:** ^1^General Surgery Department, Spedali Civili, Chirurgia Generale 3, Piazzale Spedali Civili, 25121 Brescia, Italy; ^2^General Surgery Department, Papa Giovanni XXIII Hospital, 42121 Bergamo, Italy; ^3^Emergency Surgery Department, Ospedale Maggiore, 43121 Parma, Italy

## Abstract

Gastric cancer (GC) is the fourth most common cancer and the second leading cause of cancer death in the world; 53–60% of patients show disease progression and die of peritoneal carcinomatosis (PC). PC of gastric origin has an extremely inauspicious prognosis with a median survival estimate at 1–3 months. Different studies presented contrasting data about survival rates; however, all agreed with the necessity of a complete cytoreduction to improve survival. Hyperthermic intraperitoneal chemotherapy (HIPEC) has an adjuvant role in preventing peritoneal recurrences. A multidisciplinary approach should be empowered: the association of neoadjuvant intraperitoneal and systemic chemotherapy (NIPS), cytoreductive surgery (CRS), HIPEC, and early postoperative intraperitoneal chemotherapy (EPIC) could increase the rate of completeness of cytoreduction (CC) and consequently survival rates, especially in patients with Peritoneal Cancer Index (PCI) ≤6. Neoadjuvant chemotherapy may improve survival also in PC from GC and adjuvant chemotherapy could prevent recurrence. In the last decade an interesting new drug, called Catumaxomab, has been developed in Germany. Two studies showed that this drug seems to improve progression-free survival in patients with GC; however, final results for both studies have still to be published.

## 1. Introduction

Gastric cancer (GC) is the fourth most common cancer and the second leading cause of cancer death in the world [1, 2]. The principal risk factors in development of GC are helicobacter pylori infection, atrophic gastritis, intestinal metaplasia, dysplasia, male gender, cigarette smoking, partial gastrectomy, Menetrier's disease, and genetic factors [3].

Global incidence of primary tumour locations and the histological types are constantly changing: in United States and in Western Europe the incidence of esophagogastric junction (Barrett's type) and gastric cardia adenocarcinoma is increasing [4] while there has been a reduction of incidence of distal GC since the 1970s, especially in Western countries [5].

Although GC mortality has been reduced, it remains a disease with poor prognosis and high mortality, second only to lung tumour. The prognosis of GC depends on stage and location: proximal gastric tumours (i.e., cardia tumor) have poorer prognosis compared to those in the pyloric antrum and when the disease is confined to the stomach mucosa, 5-year survival is near to 95%, while the reported 5-year survival rate for advanced GC varies from 10 to 20% [5].

Metastatic dissemination in GC may occur through the hematic torrent or by dissemination to the peritoneal cavity; this last condition is called peritoneal carcinomatosis (PC) [6], and it is considered a stage IV of GC. Recent studies show that peritoneal dissemination is more frequent than hematogenous metastases. Only 40% of GC deaths have hepatic metastases, while in 53–60% disease evolves through PC [3].

## 2. Epidemiology of Peritoneal Carcinomatosis

PC is considered the end stage of primary peritoneal malignant disorders (such as peritoneal mesothelioma) and a common manifestation of digestive-tract and gynaecological advanced cancers (such as appendicle tumour, ovarian cancer, colorectal cancer, or gastric cancer). It is generally associated with a poor prognosis; patients with PC of gastric origin have an extremely bad prognosis with a median survival estimate at 1–3 months [3, 14].

Data from the literature show that 15% of patients present PC *ab initio* and 35% of patients die of intraperitoneal recurrence for PC confined exclusively into the peritoneum [15]. Systemic chemotherapy improves median survival in metastatic gastric cancer to 7–10 months [16], but in patients with PC from GC the same improvement has not been reported [17].

Currently, at the intraoperative abdominal examination, peritoneal seeding is found in 10–20% of patients scheduled for potentially curative resection and in 40% of those at stage II-III [15, 18, 19]; 20–50% of patients treated with radical surgery will develop postoperatory peritoneal recurrence [20], and intraperitoneal spread of tumour cells is observed in 54% of patients who died of recurrence after surgery in advanced GC [21].

During the last 30 years multimodal therapeutic approaches on PC improved, resulting in a modified role of surgery: not a simple debulking operation anymore but a complete tumour cytoreduction with no macroscopic residual disease.

Sugarbaker investigated the synergism of the effects of hyperthermia and intraperitoneal anticancer chemotherapy against tumour cells; he found the existence of PC originating by low grade malignancy tumours without invasion capacity (like pseudomyxoma peritonei) that can be treated with cytoreductive surgery (CRS) and hyperthermic intraperitoneal chemotherapy (HIPEC).

In 1995 Sugarbaker definitively codified, in terms of rationale and surgical technique, the procedure of peritonectomy [22]. Following these innovative studies, a growing number of authors have been investigating this procedure [19]. Furthermore, same authors start to test these techniques in more aggressive tumours.

## 3. Pathophysiology of Peritoneal Carcinomatosis

Peritoneal dissemination of free cancer cells happens through exfoliation and leads to direct invasion of the serosa. Surgical manipulation or trauma can facilitate the mechanism [19]. Tumour cells can also diffuse passing through the “stomata”: big communicating orifices present in the peritoneal surface, between peritoneal cavity and lymphatic vessels [15]. Cells distribution into peritoneal cavity is also conditioned by physical factors: tumour primary site, effects of gravity, presence or presence of fluids (ascites, mucus, etc.), and intrinsic biological aggressiveness [23–25]. Some studies showed that there are tumours with a distinct capability to give peritoneal metastases, without giving distant metastases.

## 4. Diagnosis of Peritoneal Carcinomatosis

For preoperative diagnosis of PC, useful imaging techniques are ultrasound (US), computerized tomography (CT), magnetic resonance imaging (MRI), and 18F-Fluorodeoxyglucose Positron Emission Tomography-CT (FDG PET-CT) [15], but all these imaging techniques have major limitations in diagnosing PC because of the low-volume density of peritoneal nodules. CT and MRI are important mainly in evaluating unresectable disease and cancer staging [26, 27]. PET-CT seem to be a good option, but are expensive and have drawbacks for lesions smaller than 5 mm in diameter [14]. Concerning PC from GC, Yang et al. [28] report an accuracy of PET-CT of 87%, with a sensitivity and specificity of 72.7% and 93.6%, respectively, with a sensitivity better than CT, while for primary GC and lymph node metastases the accuracy for PET-CT is 54%. CT is not accurate (8–17% of sensitivity) particularly for malignant granulations less than 5 mm in diameter and for small bowel nodulations.

Due to the low accuracy given by the imaging, the main diagnosing methods currently used to evaluate peritoneal surface are diagnostic laparoscopy or laparotomy and peritoneal cytological examination that show a greater accuracy in diagnosing PC [14]. Diagnostic laparoscopy, with or without peritoneal washing for malignant cells, has only a III B degree of recommendation; it is used to exclude metastatic disease in tumours that are considered potentially resectable [3]. A standardized technique minimizes the risk of tumour contamination of the trocars insertion sites; this method, compared with laparotomy, is also free from risks related to the complications of diagnostic laparotomy [15] and allows staging before and after CRS + HIPEC and during the follow-up. Moreover, exploratory laparoscopy could be used in order to evaluate the efficacy of neoadjuvant chemotherapy [3].

The three main different scoring systems for intraperitoneal cancer dissemination that have been published until today are as follows.

- Japanese rules of GC [14, 29] is a classification into five categories that only considers the presence of cancerous implants and/or of malignant cells in the peritoneal washing fluid, without considering the size of malignant nodules.
- Gilly staging system for PC [14, 19], also called the Lyon score, is based on the size and distribution of malignant granulations (localized or diffused). It demonstrates that the use of complete (R0-R1) or incomplete (R2) cytoreduction, in order to assess the entirety of surgical clearance of cancer, is successful. It is difficult to confirm a R0 resection in patients with carcinomatosis. R0 and R1 can be grouped together as the outcome of these two groups is very similar. This system was also revealed to be an important prognostic indicator, as the median survival of patients with stage I or II is significantly higher than those with stage III or IV, and they can be candidates for CRS and HIPEC. However, this system does not clearly indicate the potential resectability of PC [5].
- Jacquet and Sugarbaker Peritoneal Cancer Index (PCI) [30] is based on quantitative distribution and size of peritoneal nodules. The abdomen cavity is divided into 13 regions and the lesion size is scored in each region. After a meticulous intraoperative inspection, the extent of the disease can be summed as a numerical score (0 to 39). The PCI has a prognostic value, allowing for an estimate of the probability of complete cytoreduction; it is the only method that shows in detail the nodules localization.

The completeness of cytoreduction (CCR) could be evaluated using the Sugarbaker and the Lyon scores combined, as they indicate a direct relation between CCR, prognosis, and survival.

## 5. Rationale and Technique of Cytoreduction and HIPEC

Pharmacokinetic and peritoneal permeability studies demonstrate a higher intraperitoneal concentration of drugs with chemotherapy administered intraperitoneally than with systemic administration [23, 24]. The peritoneal plasma barrier maintains a positive gradient of chemotherapy in the peritoneum, increasing the local effects of the drugs and reducing the systemic toxicity [3]. Moreover, when chemotherapy treatment is associated with hyperthermia, the locoregional effects are considerably extended, with an increased penetration up to 3–6 mm into malignant nodules and an increased antimitotic effect. Several studies confirm that hyperthermia (42-43°C) enhances the effects of antitumoral drugs, especially of oxaliplatin, mitomycin C, doxorubicin, cisplatin, paclitaxel, and irinotecan [19], also increasing the chemosensibility of neoplastic cells. The intraabdominal temperature, however, should not exceed the temperature of 43°C, in order to avoid the risk of bowel perforation [12].

According to surgical-oncologic principles, the treatment of non-metastatic GC consists of resection with total gastrectomy and D1 and/or D2 lymphadenectomy [3]. Different trials proposed multiple chemotherapy protocols using drugs like Epirubicine, Cisplatin and 5-Fluorouracil (ECF), or Epirubicin, Cisplatin and Capecitabina (ECX), that do not need a central venous access device [3].

S-1, a new drug recently introduced in Japan, combined with Cisplatinum, Paclitaxel, Docetaxel or Irinotecan has become the standard treatment for PC from GC [14].

## 6. Cytoreductive Surgery and HIPEC for Advanced Gastric Cancer

Correct radiological, clinical, and cytological stadiation is essential requirement for a better prognosis after HIPEC in GC. It is necessary to distinguish PC in early or advanced GC from PC as a recurrence of already operated GC. In fact, in the former case it is easier to succeed with a complete cytoreduction (CCR-0, R0), while in the latter, previous surgical treatment and adhesion development decrease the possibility to achieve a complete cytoreduction.

Common contraindications for HIPEC are age >70 yrs, important comorbidities, clinical aggravation with systemic chemotherapy, malnutrition, extra abdominal metastases, liver metastases when unresectable, and massive retroperitoneal bulk disease or lymph node involvement. Other minor exclusion criteria are Body Mass Index (BMI) >40, history of pelvic irradiation, carcinomatosis extended at the CT or clinically significant, more than 4 surgical procedures, occlusion, and no drop markers with neoadjuvant chemotherapy [19].

Different studies presented contrasting data about survival rates; however, they all agreed with the necessity of a complete (Tables 1 and 2) cytoreduction to improve survival. HIPEC has an adjuvant role to prevent peritoneal recurrences [19]. Gill et al. show that in patients with a CC (completeness of cytoreduction) score of 0 or 1 overall median survival was 15 months [10] (versus 7.9 months in patients with CC 2 score), with an overall mortality rate of 4.8%.

Yonemura et al. [8] in an RCT of 139 patients with T2-4 GC randomized into 3 groups (HIPEC and surgery, intraperitoneal normothermic chemotherapy plus surgery, and surgery alone) show that in the first group the survival rate was significantly higher (61% versus 43% and 42% of the other two groups), particularly in patients with serosal infiltration and lymph node positive metastases. Similar results were published by Fujimoto et al. [21] in stage II-III GC patients, and by Kim and Bae [7] in patients affected by stage III and IV GC treated with HIPEC + CRS versus surgery alone.

Kim and Bae [7] analysed 103 patients with GC stage III-IV: 51 underwent surgical resection alone and 52 received surgery plus HIPEC. Mitomycin-C at 44°C was used in the HIPEC group as intraperitoneal chemotherapy. The 5 years overall survival rate in the 103 patients was 29.97%. It was higher but not statistically significant in the HIPEC + CRS group (32.7% versus 27.1% control group). The difference, considering exclusively the survival rate of the 65 patients with stage III GC, was statistically significant (58.6% versus 44.4% control group).

In a retrospective multicentric French study undertaken between February 1989 and August 2007, Glehen et al. [10] evaluated 159 patients that underwent cytoreductive surgery and perioperative intraperitoneal chemotherapy (HIPEC and EPIC or both) and showed 1-, 3-, and 5-year survival rates of 43%, 18%, and 13%, respectively, that increase up to 61%, 30%, and 23%, respectively, in patients with a complete cytoreduction. Thanks to multivariate analysis, the authors reported the completeness of cytoreduction as being the principal independent prognostic factor. In order to correctly execute cytoreduction, the staging system should be corroborated by PCI assessment. The study showed that if cytoreductive surgery does not allow a sufficient downstaging, particularly in HIPEC, the survival rates are poor (median survival of 6–8 months).

Three recent meta-analysis [11–13] of RCTs, assessing patients with GC (with or without PC), demonstrates the survival benefit offered by HIPEC. Already in 2007, Yan et al. [11] conducted 13 studies on 1648 patients and demonstrated the positive effects in terms of overall survival rates, when adding HIPEC or HIPEC with EPIC to surgery. These studies also showed how these procedures can be complementary to adjuvant systemic treatment. The efficacy of normothermic intraperitoneal chemotherapy (NIIC) is marginal. Jin-Yu et al. [12] analyzed 15 RCTs and demonstrated that HIPEC and NIIC should be recommended in treating patients with GC, as they significantly improve the overall survival rates. HIPEC results are better, but NIIC's are still statistically significant [8]. This meta-analysis demonstrated that adding postoperative intraperitoneal chemotherapy (PIC) to HIPEC has no additional effect on overall survival rates but it improves costs and toxicity. This study also showed that intraperitoneal chemotherapy (IPC) has no effect on prevention of lymph metastasis, but could decrease by 73% the rate of hepatic metastasis. Authors demonstrated that IPC does not increase perioperative mortality and postoperative anastomotic leaks, ileus, or bowel perforation rates, but it increases the risk of marrow depression, intra-abdominal abscess, and fever. The same results are also confirmed by Sun et al. [13].

In the last decade an interesting new drug, called Catumaxomab, has been developed in Germany [31]. The Catumaxomab is a rat-mouse hybrid monoclonal antibody that is now used for patients with malignant ascites in phase II/III randomized trial. Two studies [32, 33] demonstrate that this drug seems to improve progression-free survival in patients with GC (median 71 versus 44 days; *P* = 0.03) and that it seems to improve the survival in gastrointestinal EpCAM+ tumours (EpCAM: antiepithelial cell adhesion molecule) in intraperitoneal use. However, final results of both studies have yet to be published.

CRS and HIPEC are associated with significant morbidity and mortality, also in high volume centers, and reported rates are included between 0.9–5.8% and 12–52%, respectively [34]. The main postoperative complications after CRS and HIPEC are intra-abdominal abscess, gastric or small intestinal perforation, postoperative ileus, anastomotic leakage, postoperative bleeding, fistula, sepsis, respiratory distress, hematologic toxicity, and urinary disturbance [6, 19]. In the same group of patients, the main causes of death include anastomotic leakage, sepsis, postoperative bleeding, intestinal fistula, and disseminated intravascular coagulation (DIC) [35].

The aggressiveness of GC disease is the main cause of this unfavourable prognosis. Recently, Yonemura et al. [9, 14] proposed a multimodal strategy that associates neoadjuvant intraperitoneal and systemic chemotherapy (NIPS), CRS + HIPEC and EPIC. The rationale of this method is to reduce tumour burden before surgery with NIPS, a bidirectional chemotherapy that attacks PC from both sides of peritoneum (from the peritoneal cavity and from subperitoneal blood vessels), together with CRS and HIPEC, in order to reduce macroscopic and microscopic PC. The aim of EPIC is then to eradicate residual intraperitoneal cancer cells before the development of adhesions.

The NIPS technique is characterized by a first phase in which patients are treated with 60 mg/m^2^ of oral S-1 for 21 days, followed by one week of rest. A port system has been previously placed into the abdominal cavity under local anesthesia, with the tip in the Douglas pouch. On the 1st, 8th, and 15th postoperative days, 30 mg/m^2^ of Taxotere and 30 mg/m^2^ of Cisplatinum with 500 mL of saline are infused into the peritoneal cavity. Authors recommend two cycles of treatment to achieve a negative cytology status. Complications after NIPS have been reported in 4 out of 79 patients (1 with grade 4 of bone marrow toxicity, 3 with a renal dysfunction). In 3 patients, infections around the periportal space, that led to the port remotion, were reported. This study shows a washing cytology negativization in 41 out of 79 patients (63%) [4].

Also Glehen et al. [10], in a retrospective multicentre study, recommend the routine use of neoadjuvant chemotherapy to improve surgical results and to exclude patients who do not respond to the therapy form HIPEC treatment.

## 7. Conclusions

The aforementioned procedures should be exclusively performed in highly experienced centres because of the special surgical expertise needed to achieve high rates of complete cytoreduction [14, 19].

Patient selection is very crucial and should be carried out by a multidisciplinary group of specialists (anaesthetists, surgeons, clinicians, and oncologists) in order to achieve better results and to reduce the high costs related to these procedures and relevant complications.

An interdisciplinary approach should be developed further: the association of NIPS, CRS, HIPEC, and EPIC could increase the rate of CC-0 procedures and consequently survival rates, particularly for PCI ≤6 [5, 11, 21, 32]. Neoadjuvant chemotherapy, routinely recommended for management of GC without PC, may improve survival also in PC from GC [10, 34–37] and adjuvant chemotherapy could prevent recurrence from GC [10]. Finally, the study of molecular and serum tumour markers could provide valuable prognostic information and would allow for a better selection of subsequent treatment combinations [14, 38].

## Figures and Tables

**Table 1:** Comparing main studies in terms of median survival, 5-year survival rates, and morbidity.

Authors	Year published	Type of study	Stage of GC	Number of patients	Type of protocol	Median survival (months)	5-year survival rates	Morbidity
Fujimoto et al. [21]	1999	RCT	Stage II-III	141	HIPEC + surgery versus surgery alone	N/A	76% versus 57%	N/A
Kim and Bae [7]	2001	PCS	Stage III-IV	103	HIPEC + CRS versus surgery alone	36 versus 22.9	32.7% versus 27.1%	33.2% versus 36.5%
Yonemura et al. [8]	2001	RCT	Stage II-III	139	HIPEC + surgery versus NIC + surgery versus surgery alone	N/A	61% versus 43% versus 42%	4% versus 0% versus 4%
Yonemura et al. [9]	2009	PCS	Stage III-IV	79	NIPS versus NIPS + surgery	N/A	20.4% versus 40%*	11.4%
Glehen et al. [10]	2010	RCS	Stage III-IV	159	HIPEC + EPIC versus EPIC HIPEC	9.2 (overall)	13% (overall)	27.8% (overall)

HIPEC: hyperthermic intraperitoneal chemotherapy; EPIC: early postoperative intraperitoneal chemotherapy; PIC: perioperative intraperitoneal chemotherapy; NIC: normothermic intraperitoneal chemotherapy; NIPS: neoadjuvant intraperitoneal and systemic chemotherapy; NIIC: normothermic Intraoperative Intraperitoneal Chemotherapy; DPIC: Delayed Postoperative Intraperitoneal Chemotherapy; RCS: Retrospective Case Series; PCS: Prospective cohort study; PCS: prospective case series; N/A: not available.

*2-year survival rates.

**Table 2:** Comparing main meta-analysis in terms of overall survival and morbidity.

Authors	Year published	Type of study	Stage of GC	Number of patients	Type of protocol	Overall survival	Peritoneal recurrence	Mortality and morbidity
Yan et al. [11]	2007	Meta-analysis (13 RCTs)	Stage I–IV	1648	Surgery + HIPEC/HIPEC + EPIC/NIIC versus surgery alone ± systemic chemotherapy	HR = 0.60^1^ HR = 0.45^2^ HR = 0.67^3^	RR = 0.84^§^ RR = 0.51 RR = 1.00^§^	RR = 1.43 RR = 1.01/1.81/0.76/2.37/4.33^#^
Jin-Yu et al. [12]	2012	Meta-analysis (15 RCTs)	N/A	1713	HIPEC versus HIPEC + PIC versus NIIC	HR = 0.60 HR = 0.47 HR = 0.70	OR = 0.69	OR = 2.29 OR = 1.04/2.60/1.61/0.39/5.74/3.67/3.57^$^
Sun et al. [13]	2012	Meta-analysis (10 RCTs)	Stage II-III	1062	HIPEC* versus surgery	MMC RR = 0.75 5-FU RR = 0.69	RR = 0.45	N/A for Mortality RR = 1.68/0.52/1.38/0.79/1.47^*£*^

HR: hazard ratio; RR: risk ratio; OR: odds ratio; MMC: mitomycin C; 5-FU: 5-fluorouracil; HIPEC: hyperthermic intraperitoneal chemotherapy; EPIC: early postoperative intraperitoneal chemotherapy; PIC: perioperative intraperitoneal chemotherapy; NIC: NIIC: normothermic intraoperative intraperitoneal chemotherapy; RCS: retrospective case series; PCS: prospective case series; N/A: not available; PC: peritoneal carcinomatosis.

^1,2,3^HIPEC, HIPEC + EPIC, and NIIC, respectively.

^§^No differences in morbidity between HIPEC and NIIC groups and the respective control arms.

^#^Morbidity of anastomotic leak, bowel fistula, pancreatic fistula, intra-abdominal abscess, and neutropenia, respectively.

*Data calculated with risk ratio (RR) and divided in two subgroups for analysis: mitomycin C subgroup RR = 0.75, 5-Fluorouracil RR = 0.69 versus control group RR = 0.45.

^*£*^Morbidity of bone marrow suppression, anastomotic leak, bowel fistula, adhesive ileus, and liver disfunction, respectively.

^$^Morbidity of anastomotic leak, ileus, bowel perforation, pancreatic fistula, marrow depression, fever, and intra-abdominal abscess, respectively.
